# Perception and Cognition Are Largely Independent, but Still Affect Each Other in Systematic Ways: Arguments from Evolution and the Consciousness-Attention Dissociation

**DOI:** 10.3389/fpsyg.2017.00040

**Published:** 2017-01-24

**Authors:** Carlos Montemayor, Harry H. Haladjian

**Affiliations:** ^1^Department of Philosophy, San Francisco State UniversitySan Francisco, CA, USA; ^2^Laboratoire Psychologie de la Perception, CNRS, Université Paris DescartesParis, France

**Keywords:** cognitive penetrability, consciousness, visual attention, evolution, dissociation, language, concept acquisition

## Abstract

The main thesis of this paper is that two prevailing theories about cognitive penetration are too extreme, namely, the view that cognitive penetration is pervasive and the view that there is a sharp and fundamental distinction between cognition and perception, which precludes any type of cognitive penetration. These opposite views have clear merits and empirical support. To eliminate this puzzling situation, we present an alternative theoretical approach that incorporates the merits of these views into a broader and more nuanced explanatory framework. A key argument we present in favor of this framework concerns the evolution of intentionality and perceptual capacities. An implication of this argument is that cases of cognitive penetration must have evolved more recently and that this is compatible with the cognitive impenetrability of early perceptual stages of processing information. A theoretical approach that explains why this should be the case is the *consciousness and attention dissociation* framework. The paper discusses why concepts, particularly issues concerning concept acquisition, play an important role in the interaction between perception and cognition.

## Introduction: Evolutionary Arguments for a Perception and Cognition Interface

This paper critically assesses the view that there are systematic and robust influences from cognition on perception at the early stages of processing, which could be considered cases of cognitive penetration. While we agree with the criticisms that there are empirical “pitfalls” in the experiments allegedly reporting cognitive penetration (see Firestone and Scholl, 2016), there also are difficulties regarding the view that there is a sharp distinction between perception (the processing of sensory information that occurs at several levels) and cognition (the judging of representational contents related to reasoning). Besides being problematic theoretically, the assumption that a sharp distinction between all cognition and all perception must be an essential aspect of the mind may even be empirically false. The criticisms around the notion of penetrability need to be more balanced so that it accounts for an architecture consisting of some cognitively impenetrable modules (characteristic of early perception) along with others that are susceptible to top-down influences (characteristic of late perception). Such varied effects must be available in perception to understand abilities such as predictive coding and conceptual attention.

We focus on concept acquisition to explain the interface between cognitively penetrable perception and cognitively *im*penetrable perception, and particularly on the fact that concept acquisition is also a perceptual, rather than a strictly cognitive process involving only reasoning or judgment. Even if the brain’s architecture is organized in a modular and encapsulated way, there can still be a conceptual *interface* between perception and cognition. It is at this conceptual interface, which is also responsible for explicit or discursive judgment and inference, where most interactions between perception and cognition will occur that can contain instances of cognitive penetration. We will explore the issue of concept acquisition at different stages of processing and explain how it relates to top-down pre-cueing. This relation will reinforce our point that a balanced combination of any possible cognitive penetrability and early impenetrability is critical. In fact, we aim to show that conceptual interfaces between cognition and perception are crucial for understanding how our species developed sophisticated forms of attention.

One approach to achieve this balanced interface perspective is based on the consciousness and attention dissociation (CAD) framework (Montemayor and Haladjian, 2015). This framework characterizes the relationship between consciousness and attention, and claims that attention is significantly dissociated from consciousness, with different levels of interactions between attention and conscious awareness. This distinction is important because there is abundant evidence of cognitive effects on attention without conscious awareness—an unconscious form of cognitive guidance—as well as cases in which motivational states guide implicitly, sometimes against the conscious judgments of subjects, as in cases of implicit bias (see Montemayor and Haladjian, 2015, for a discussion of the evidence in vision). These cases of guidance and selection in perception may be conceived as attention routines, and many will be independent, and even disagree, with conscious perception. Crucially, for the topic of cognitive penetration, CAD allows for the systematic guidance of late perception by cognitively driven attention, while also allowing for the cognitive impenetrability of early perception.

These different types of guidance and influence on late perception (voluntary and involuntary, conscious and unconscious) help clarify some problems concerning extant discussions on cognitive penetration. Some alleged cases of cognitive penetration may readily be excluded, for instance cases of explicit voluntary judgment on perceptual contents that are not even indirectly influenced by beliefs or discursive inference. Some motivational and emotional forms of guidance are more problematic, as they typically occur independently of explicit propositional attitudes, although they can easily be understood as part of the attentional guidance on late perception. But it seems that if all implicit forms of motivational and cognitive guidance are excluded, as defended by the sharp delineation view, then it is too easy to conclude that perception is never penetrable by cognition. We will resist this conclusion by arguing that it is an implausible view of the complexity of perception—and of its *evolution*. We will also argue against the opposite view that cognitive penetration is widespread, as some proponents of cognitive penetrability propose. Some forms of perception, specifically early perceptual processing, must be impenetrable. The problem is one of balance: there must be systematic forms of influence on perception without major disturbances to the evolutionarily developed and required perceptual invariances for successful navigation and motor control. The dissociation between consciousness and attention provides this more nuanced theoretical approach, and it advances the debate beyond the strict dichotomy between cognition and perception.

In addition, the CAD framework is particularly well suited to address cognitive penetration because it is supported by a vast amount of findings, specifically in vision science (Montemayor and Haladjian, 2015). The ‘early versus late perception’ distinction was introduced in order to interpret findings in vision science. Early visual perception includes sensory processes that are specialized for handling specific types of information used in constructing representations independently of beliefs (Pylyshyn, 1999). Late perception involves selective processing by top-down attention and other cognitive processes (Raftopoulos, 2015b). Just like this distinction generalizes to other perceptual modalities and to the more general distinction between cognition and perception, CAD also generalizes to all kinds of dissociations between subjectively conscious experience and attention routines that do not necessitate conscious awareness, including emotions and memory. The central tenet of the CAD framework is that there must be some dissociation between attention and phenomenal consciousness (subjective experience) with some extant theories indicating a high degree of dissociation. Thus, CAD is a framework to better understand, model, and integrate findings and theories on consciousness and attention based on how they are dissociated from each other. In this paper we present the implications of CAD for the topic of cognitive penetration.

The crucial argument we make in support of these claims concerns evolution. Also based on the CAD framework, the argument is as follows.

(a) Perceptual systems evolved from basic to complex forms of processing, and some are less cognitively penetrable than others (e.g., early perceptual stages are cognitively impenetrable).
(b) Perceptual states produced by such systems can be defined in terms of intentionality (the way in which mental representations are *about* things and features in the world): perception is always about features of the environment that can in principle be interpreted propositionally (although they need not be propositional to be intentional).
(c) If perceptual systems evolved, then intentionality also evolved.

Therefore, some forms of intentionality are more cognitively penetrable than others, and an *interface for penetrability* is needed for concept acquisition and global access (including access to propositional content).

This argument shows why evolution matters to the debate on cognitive penetration, and why penetrability is more complicated than previously thought. CAD can help explain the relationship between cognition and perception, and indicate where cases of penetrability may occur. For instance, one possibility is that there may need to be two interfaces between cognition and perception, one concerning phenomenally conscious experiences and another concerning non-phenomenally conscious perceptual contents. Such interfaces will be critical for all kinds of conceptual and pre-conceptual learning that guide attention routines.

A discussion about what is meant by ‘cognitive penetration’ is required to fully understand the implications of this argument. By ‘cognitive penetration’ most authors intend a general category of cognitive influences on how perceptual information is processed by sensory mechanisms, which includes cases in which the beliefs and desires of perceivers somehow determine what they perceive. This of course can be interpreted in many ways. The demarcation between cognitively penetrable and impenetrable perception was originally proposed to understand cognitive architecture, but it now encompasses cases in which top-down attention influences bottom-up early attention routines, independently of specific commitments regarding architecture (Vetter and Newen, 2014). As mentioned, views at one end of the possible degrees of penetrability deny that cognitive penetration captures a truly unique type of influence of cognition on perceptual processing (e.g., Firestone and Scholl, 2016). Such views would never consider systematic influences of cognition on perception as legitimate cases of cognitive penetration. On the other hand, views that state that there is no boundary between cognition and perception deny that cognition could be dissociated from perception (e.g., Clark, 2013).

Thus, a critical issue is how to clearly specify legitimate cases of cognitive penetration—cases in which the influence of cognition on perception is not trivial or easily explained by appeal to inference (Firestone and Scholl, 2016), or some other cognitive process such as judgment or interpretation. This becomes especially important when authors arguing for the case of penetrability do this by giving examples of changes to higher levels in perception, those that are beyond the initial stages of sensory processing. For example, some findings indicate that throughout the stages of perceptual processing there are both forward and backward neural projections that contribute to perception (e.g., Vetter and Newen, 2014). Yet, these do not necessarily indicate that early perception is penetrable by cognition. We argue that the more interesting cases of cognitive penetration would not be at the higher level of perceptual judgment or the interpretation of the output from sensory processing. Nor would they be cases where voluntary attention simply changes the perceptual *stimulus* or input (e.g., looking to the left based on my desire to change my gaze should not count as a case of cognitive penetration). Radical cases of penetrability would influence perceptual processing directly at early stages, and not simply at a higher attentive (or cognitive) level.

More specifically, the most problematic form of cognitive penetration would have to occur at the level of processing called ‘early vision’ or early *perception* more generally (see Pylyshyn, 1999). Instances of radical cognitive penetrability should show that perception, particularly early perception, cannot “resist” the influence of content coming from inferences, beliefs, or desires. This could happen quite selectively: not all beliefs and desires can directly affect perception, but only some specific ones in specific situations. What is crucial is that if radical cognitive penetration exists, then there is the possibility of causal influences from cognition that directly modify perception, even when all else is being equal at the sensory input level, including how attention is being allocated. This causal influence must explain directly how early perception is processed—otherwise, purely conceptual influences could explain cognitive penetration (see Raftopoulos, 2014, pp. 605–606 for discussion). We shall argue against this radical form of cognitive penetration.

Cognitive penetration is a crucial topic in philosophy of perception because of how it relates to controversial issues in epistemology or the theory of knowledge. For instance, there is the view that the contents of perception are propositional (i.e., they have truth conditions, just like the propositions expressed by sentences), and that perception is akin to belief—a kind of *propositional attitude* (Byrne, 2005). There is also the view that perception need not have propositional content (Crane, 2009). This issue is clearly related to the topic of non-conceptual content in perception. In these debates, it is generally taken for granted that the focus of analysis is perceptual conscious experience. But CAD shows this is an assumption that should not be taken for granted because what is true about phenomenally conscious perception need not be true about perception in general—there are types of non-phenomenally conscious perception as in blindsight (e.g., see Kentridge, 2011). More important, CAD explains why these apparently opposite views could be true about different types of perception—one cognitively penetrable at the propositional, later perceptual level and the other cognitively impenetrable at the non-conceptual, early perceptual level. As we argue below, this is actually a consequence of the argument from evolution.

To illustrate the importance of CAD to understand different types of cognitive impenetrability, consider the most basic kind of conscious experience, for instance of color. One possibility CAD allows for is that early color perception is experienced in the exact same way as in other organisms that lack the top-down routines dependent on cognitive capacities. This possibility plays a major role in motivating the notion of phenomenal consciousness, particularly for “first order” theorists, who deny that experiences must be part of a thought or representation for them to be conscious. This approach suggests that many species, certainly mammals, must have phenomenal experiences that are analogous to human phenomenal consciousness. For such overlap in experiences of color, it seems necessary to adopt the view that early vision color is impenetrable (for dissent see Macpherson, 2012). So what about color perception that is processed at the interface with working memory, conceptual categorization, and motivational guidance (e.g., perceptually judging the typical color of an object or evaluating the beauty of a combination of colors)? At this level, it is clear that color perception would be susceptible to different kinds of top-down effects, and these could count as cognitive penetration at later stages of processing. In humans, these two types of perceptual processing come apart, and only CAD makes sense of this possibility: conscious early (bottom-up) vision without top-down attention modulation and conceptualized color detection, susceptible to cognitive and motivational modulation. An intriguing possibility, entailed by the argument from evolution, is that some animals experience color in a modular, and more encapsulated way, because they lack the conceptual interfaces required for late perceptual modulation and judgment.

The consciousness and attention dissociation thus helps us understand the cognitive impenetrability of early perceptual processes, without maintaining that there is no room for cognitive penetrability at more integrated levels of perception and cognition, in a way that generates an interaction between these levels. It also facilitates the theoretical characterization of cognitive influences on unconscious perception that play no role in conscious experience, and vice versa. Combined with the argument from evolution, CAD justifies the impenetrability of early perception based on the importance of perceptual invariances to navigate the environment, for example, which must have evolved early on, independently of cognitive and motivational influences. It is precisely because different kinds of intentionality evolved at different times that there must be interfaces between perception and cognition, some of which need not be fully fledged conceptual inference. This is why processes involved in concept acquisition are relevant for striking a balance between the ‘pervasive cognitive penetration’ and ‘no cognitive penetration’ views.

Like any theoretical category, that of ‘early vision’ (which can be extended to early *perception*) has fuzzy boundaries. There is agreement, however, that early vision must include modularly specific (cognitively impenetrable) feature detection, such as color, motion, or orientation, typically before the involvement of working memory. It may also involve objecthood, without the cognitive imprint of conceptual categories. One may say that at the very first stages of perception, there is sentience of phenomenally experienced features, structured spatially and temporally, which can be cross-modally integrated by feature maps. This processing must preserve external invariances concerning light reflectance, shape, distance, and duration (among many other invariances that allow for reliably accurate navigation and coordinated motor control). In this sense, perceptual invariances are preserved by cognitive impenetrability from motivational and conceptual attention modulation (at least in humans). The later involvement of working memory allows for such cognitive and emotional modulation, and what was consciously experienced without the imprint of categorization is now experienced under a conceptual or motivational influence or category. This cognitive transition has implications for how to understand perception in other species and also with respect to the evolution of our own perceptual system. This is one of the reasons why CAD and the argument from evolution must inform our understanding of cognitive penetration.

Based on these considerations, it seems that there are two kinds of cognitive impenetrability: phenomenally conscious (basic feature perception) and non-phenomenally conscious (feature detection outside of awareness). Likewise, there might be two kinds of cognitive penetrability, one phenomenal (motivational influences on perception) and the other non-phenomenal (conceptual influences in blindsight-like detection). Once conceptual capacities are in the picture, however, one can always *interpret* perceptual contents by providing a propositional explanation or interpretation. Consider the contrast between explaining and directly causing the contents of perception. In typical cases of automatic or effortless inference, you can infer that someone is late by looking at their facial expression or how they are looking at their watch, but this does not mean that you are seeing “lateness.” Emotion perception is more complicated, but it might be susceptible to similar interpretative treatments (for dissent, see Siegel, 2006; Newen and Vetter, 2017). We can infer someone’s joy through their facial expressions, but we do not necessarily *see* the actual feeling of joy. In this sense, inference can influence what someone perceives without changing radically how the visual system perceives environmental features, which would remain impenetrable. What causes the contents of perception at early stages remains untouched by top-down modulation.

Such inferential influences could be implicit and not depend on any kind of voluntary guidance. The notion of ‘inference’ is flexible enough that it could occur at all stages of perceptual predictive processing in perception (see Clark, 2013), where such processing can be influenced by the statistical properties of experiences or contexts (e.g., see Yuille and Kersten, 2006). This more flexible notion seems to problematize the distinction between impenetrable and penetrable perception, but once the CAD framework is in place, one can argue, based partly on the argument from evolution, that early perceptual statistical processing need not be considered susceptible of any top-down influence. Such probabilistic information about perceptual properties is compatible with encapsulation (Raftopoulos, 2015a).

A critical point that deserves emphasis is that cognitive penetration should not jeopardize the stable invariances of perception. This constraint is particularly important for results that aim to show putative forms of penetrability concerning basic information for navigation, such as information concerning distance and depth. If penetration occurs in these cases, it must be shown that they are not pervasive to the degree that someone who is simply walking out of a room would be disoriented by the changes in size, distance, and depth that are based on her beliefs and desires. If cognitive penetration entailed this kind of disruption of basic perceptual invariances, then such cases of penetrability would be just as disorienting, if not more disorienting, than hallucinations. Typically, hallucinations are explained in terms of changes in physiology (e.g., a deliberate neurophysiological change caused by ingesting certain drugs), rather than simple changes in belief and desire. Thus, an important constraint is that cognitive penetration should not be conceived in ways that would entail radical alterations to perception, analogous to those caused by physiology from external sources. Perception (e.g., early vision) must preserve invariances reliably. For truly radical cognitive penetration to occur, there must be evidence that top-down conceptual information influences the early stages of visual perception beyond simply facilitating the processing of visual information (e.g., attentional effects) (Raftopoulos, 2015b).

As mentioned, another important consideration is the notion of intentionality (i.e., the way in which mental representations are about things and features in the world) and how evolution can explain it. Intentionality may be very basic, processed in a modular fashion, and responsive to immediate information from the environment, or it can be more abstract, categorical, and influenced by judgments and inferences. Various forms of intentionality will correspond to the evolutionary record of such capacities, as well as how widespread they are across species (the earlier, the more widespread). Intentionality will require a conceptual interface at some level, at least in humans, especially when faced with novel stimuli or situations that demand categorization. It is this area of conceptual development that requires scrutiny in terms of potential interfaces for cognitive penetrability of late perceptual stages of processing.

Concept acquisition of perceptual categories, we propose, is the best example of why an interface between perception and cognition is needed. Interesting cases of cognitive penetration could be defined in terms of such interfaces concerning concept acquisition, and this is the strategy we follow here. An important question is whether there are pre-cuing effects on concept acquisition. Since pre-cueing determines how attention is allocated and can change the background neural activity in a way that helps determine what is perceived, it may also determine or bias how a concept is obtained or categorized through perception. The relation between categorical reasoning and categorical capacities based on what ethologists call ‘fixed action patterns’ is one that deserves attention in this regard. A thorough evaluation of the evolution of intentionality across different species should include an examination of pre-cuing effects on these proto-conceptual intentional representations.

## Defining an Interface for Cognitive Penetration that does not Jeopardize Early Perception

A more essential starting point is to define what is meant by perception and cognition. Perception is the processing of external information by the sensory systems, such as visual or auditory information. It has various stages, and can be broadly categorized between early perception, which is comprised of encapsulated sensory processing modules (e.g., see Pylyshyn, 1999; Raftopoulos, 2015b), and late perception, which includes multi-modal integration, event perception, and object recognition (e.g., see Cavanagh, 2011). Perceptual information processing often leads to the *subjective experience of that information*, for example, of seeing an object or hearing a sound. Yet sensory processing does not need to enter conscious awareness to be perceptually registered—a lot of it can happen in the background. Importantly, perception is considered to be essentially a “belief-independent” process (particularly the early kind). A key question, notoriously difficult in epistemology, is how can such belief-independent processes justify beliefs? Again, this issue concerns the interface between perception and cognition.

When I see an apple, for example, my visual system is processing information about the features of this object, but how exactly is such processing related to the justification of my belief that I am seeing an apple? If all I perceive is shape and color then the justification of my belief is mostly independent of perception and it must be some kind of inference. But there is no problem in saying that I see an apple (or that I see an object as an apple), and that what I see justifies my beliefs because of the top-down modulation of concepts. This is compatible with the encapsulation of color and shape perception, and CAD is particularly helpful in explaining how this is possible. This helps solve the problem of how epistemically unjustified early processing gives rise to perceptually justified beliefs by the top-down influences of concepts on late perception.

Cognition involves more deliberate modulation by top-down processes, like using focused attention to search for a specific object, and includes action-planning, self-reflection, and abilities related to language. All of these processes are closely linked to consciousness and propositional content (specifically the so-called ‘access consciousness’—Block, 1995). These processes are generally epistemic, but they can also include more complex forms of cognition and conscious experience, like aesthetic and moral judgments. The implication of radical cognitive penetration is that such goal-oriented higher-level processes can directly affect the way in which information is initially processed by sensory systems such that it affects feature detection (e.g., the color of the object to be found). We shall argue that they can only alter them indirectly, by the modulation of late perception.

The question at issue is just how much can cognition affect low-level perceptual processes? Will this be a form of pre-cueing that simply directs impenetrable modules and routines, or does it actually affect the processing of perceptual information within the module (beyond attentional effects)? Is any aspect of low-level perception truly cognitively penetrable? Given the constraints mentioned above, as well as the argument from evolution, the answer is that cognitive penetration cannot be pervasive, and if it happens, it has to happen at the right level (e.g., late perception, after the intervention of at least working memory) so that *perceptual invariances* are not affected and basic abilities necessary for survival, such as navigation, are possible. To reiterate, early perception is not likely to be susceptible of any kind of cognitive penetration. One possibility, compatible with CAD, is that access conscious penetration of perception may occur without phenomenally conscious penetration on early perceptual experiences and vice versa. With respect to phenomenal consciousness, a similar distinction is unproblematic: early phenomenal conscious vision may be non-conceptual and then phenomenal concepts are deployed to categorize experiences (see Loar, 1997).

As mentioned, some authors argue that cognitive penetration *never* genuinely occurs. Instead, what falls under the category of “penetration” is judgment or cognition, and it never affects perception as such (see Firestone and Scholl, 2016). Other authors defend the view that cognitive penetration affects perception in all sorts of ways, such that belief systematically alters perception (e.g., Siegel, 2006, 2010; Stokes, 2012). This is argued to occur even at the earliest stages of processing. Given the amount of top-down influence on perceptual processing on a neural level (e.g., see Gilbert and Li, 2013; Vetter and Newen, 2014), this view is not implausible. According to this pervasive-penetrability view, our beliefs, desires, and goals affect perception in multiple ways. What we perceive, therefore, is susceptible to a vast array of cognitive influences.

The pervasive-penetrability view presents a difficult challenge. If cognitive penetration always and systematically occurs, perception would inform us almost always about what we *already* believe or feel, instead of informing us about features of the world (particularly when we encounter novel objects or events). This is a problem that is especially worrisome for epistemology (Stokes, 2012). Clearly, there would need to be *varieties* of perceptual penetration with varying degrees of penetrability. If experiences are analogous to beliefs in the sense that they require critical judgment and justification, then one must reflect on, as well as systematically analyze, what one perceives. This reflective analysis would constitute an effortful and highly top-down form of *attention* (perhaps even effortful voluntary attention to explicitly judged perceptual contents). Problematically, such a belief-based attentive process would need to dominate all other forms of perceptual attention for pervasive penetration to occur.

The consciousness and attention dissociation and the argument from evolution offer a way out of this challenge. It could be that cognitive penetration only affects *access consciousness* (i.e., access to information available for thought, memory, and action, but without subjective experience) at higher levels of cognitive integration. All effects of cognition on perceptual experiences can be explained by appeal to concepts, beliefs, or inferences, and perceptual contents remain impenetrable at the early stages. It could be, therefore, that top-down attention routines operate independently from phenomenally conscious perception. Motivational effects may be explained at higher levels of integration, which need not modify the contents of early phenomenally conscious perception. The forms of perceptual experience that evolved early, such as experiences of color, would be impenetrable. This theoretical possibility would solve the epistemic problem presented above. CAD could also explain why the pervasive penetration of conceptualized contents in access consciousness need not entail the pervasive penetration of phenomenally conscious perception (subjectively experienced perception).

There are, however, good reasons to believe that the view at the other extreme that rejects any form of penetrability is also too radical. For example, social interactions require perceptual processing and an understanding of the situational context (including other agents) in order to succeed. Categorizing new objects, events, or situations also requires a level of cognitive influence that may depend on previous experience or knowledge. The view of perception as Bayesian inference, for example, presents models of how perception can be constrained by prior experience, biasing detection of more likely features and limiting the possible interpretations of this information (e.g., see Kersten et al., 2004; Yuille and Kersten, 2006). Although we would argue that this sort of biasing is not a form of cognitive penetration of early perceptual processing, it can influence how this processing occurs and particularly influence how the contents of perception are interpreted. Such reasons exemplify why there must be an interface for cognitive penetration. These would be epistemically fundamental cases of cognitive penetration at later stages of perception, where the cognitive integration of emotion, cognition, and perception is at work. Here we try to strike a balance between these opposite views by appealing to the CAD framework and the argument from evolution (see Haladjian and Montemayor, 2015). A more nuanced view is required not only to solve the epistemic problem mentioned above, but also to achieve a comprehensive theory of perception that accounts for the epistemic and motivational significance of perception, and the Bayesian approach is particularly helpful here.

How exactly should the evolution of intentionality be understood, particularly with respect to CAD and cognitive impenetrability? One possibility is that humans and other species share many forms of early perception, with non-conceptual intentional content, which could be understood in terms of Peacocke’s (1992) account of “scenario content.” As Crane (2009) clarifies, such scenario content must be interpreted in terms of being in a *state* with non-conceptual content—a representational state such that being in it does not require the possession of concepts—even though such contents could be properly characterized by concepts by a creature with conceptual capacities, such as humans. We cannot be certain about how animals experience such contents, but it is highly likely that they must have similar experiences. Animals navigate, identify objects, react to color, and have similar sensorial systems. At some point in our evolution, our brains created routines to cognitively guide attention, but these routines cannot directly change early perception due to the requirement of feature constancy for survival, which includes features such as color and time (Lisi and Gorea, 2016). Then, even later in our evolution, we learned to explicitly interpret our perceptual experiences and to linguistically articulate such interpretations in terms of discursive inference (a capacity that seems to be exclusively human). Thus, genuine cases of cognitive penetration should not appeal to explicit inference, as when one “sees” that someone is late. But perception at higher levels of cognitive integration (e.g., above early vision) may present interesting cases of cognitive penetration by conceptualization. This would leave early processing encapsulated and impenetrable, and it would also open the door to interfaces between preconceptual perception and cognitively guided, conceptual perception.

Forms of cognitive integration also evolved, and they matter for the way in which perceptual contents are processed. For example, the cross-modal integration of information (e.g., auditory and visual) can indicate influences from one modality on another when attention is directed in a certain way (e.g., Palmer and Ramsey, 2012). Such cross-modal integration is often, though not always, related to conscious experience, with some theories of consciousness relying on the integration of information from multiple sources to produce the unified experience of consciousness (e.g., Tononi, 2012). This multi-level approach could help model possible forms of cognitive integration in terms of different interfaces that evolved at different times. Early perception remains impenetrable to guarantee stability, but in the course of evolution, contents are accessed and integrated, without affecting early perception. Then memory and motivational systems are also integrated into more complex cognitive states, guided by cognitively driven attention.

Early perceptual processes must, above all, provide reliable information about the environment independently of motivation or cognitive modulation. They include feature-based and object-based attention (Treisman, 1988), and motion tracking mechanisms (Pylyshyn, 1989; Cavanagh et al., 2001). Top-down pre-cuing and cognitive guidance operate at higher levels, after early selection mechanisms of attention have occurred (Yeh and Chen, 1999; Theeuwes, 2010). Thus, early vision provides a basic realm of perceptual experiences that inform navigation, immediate engagement with the environment, and even forms of planning that can be found in other species, such as birds (Clayton and Dickinson, 1998). As mentioned, this form of intentionality may be understood in terms of the notion of ‘scenario content’—an intentional state that need not be constituted either by concepts or propositional contents for it to be *representational*. Navigation in many species seems to demand this kind of intentionality and it must have evolved early (for discussion on how this topic relates to the distinction between analog and digital formats of mental representation, see Montemayor, 2013, chapter 3). It is very likely that in creatures with phenomenal consciousness, scenario content is deeply linked to basic experiences that inform them about the environment much in the same way as they inform us. Although many skillful reactions to the environment occur outside phenomenal consciousness, conscious experience is our most immediate guide for action. Access to content, on the other hand, requires higher levels of integration and the intervention of propositional attitudes, such as beliefs.

There is a related issue concerning how attention works outside conscious awareness in species that may not have phenomenal consciousness. Non-human species with complex attentive systems, such as dragonflies (Wiederman and O’Carroll, 2013), are also not likely to access navigational information propositionally (in terms of access consciousness and conceptual judgment). Here CAD presents an interesting possibility. Perhaps those attention capacities for navigation and object tracking in species like insects are *extensionally equivalent* to those of organisms that rely on phenomenal consciousness (they overlap in terms of their reference and how the organism reacts to stimuli). But for such extensional overlap to be possible, these early perceptual processes must be impenetrable, or at the very least, the impenetrability of such perceptual processes is the best explanation we have for their overlap across species. Obviously, understanding exactly how much perceptual guidance happens outside conscious awareness is an empirical issue. The claim we defend here is that the distinction between cognitively impenetrable perception and cognitive penetration is fundamental to account for the complexity of perception and its evolution. The challenge is to understand the relation between cognitively impenetrable perception and cognitively penetrable perception. To this end, we now proceed to discuss concept acquisition—one of the clearest instances in which an interface between cognition and perception must occur.

## Concept Acquisition

The sharp distinction between cognition and perception, which some critics of cognitive penetration theorize as a central feature of the mind (see Firestone and Scholl, 2016), confronts a particularly pressing problem at the heart of the cognitive sciences: concept acquisition. In fact, the claim that such a strict demarcation is an essential aspect of the nature of the mind may even be empirically false (Kosslyn, 1980, 1994). For our purposes, we will focus only on how the sharp demarcation between cognition and perception generates problems for the issue of concept acquisition. We aim to show that although the pervasive cognitive penetration view cannot be true, as argued above, the opposite view that claims that no cognitive penetration ever occurs is also wrong. An important clarification is that cognitive penetration can occur in late perception (after early perceptual processing), and that preconceptual processes play a major role in providing an interface between cognition and perception at that level. Thus we defend the view that early perception cannot be directly affected by cognition, but that there is an interface that makes late (penetrable) perception possible and, in fact, systematic. The main difficulty is to explain the acquisition of perceptually based concepts that are critical for basic recognition tasks.

Just as we need to be clear about the sense in which cognition determines perception, we also need to be clear about what is meant by ‘conceptual cognition.’ First, consider the distinctions between *memory, recognition*, and *seeing*. Remembering is clearly different from seeing and memory-based attentional effects. Although memory may be crucial to guide perception and categorize novel objects (e.g., Vlach, 2016), it does not *determine* what we see. But why should conceptually based recognition be on par with memory as a non-perceptual process? Take for instance the evolutionarily crucial skill of recognizing kin and enemies. This fundamental capacity seems to be part of the perceptual system, and it seems to be the result of its evolution (Millikan, 2005). Additionally, recognizing something does not always require a full perception of it, since inferential processing can use key features to inform the representation based on memory, which indicates that recognitional abilities in animals must be a combination of perceptual and preconceptual capacities. Because of how basic these skills are for survival, two forms of recognition could be postulated: one dependent on memory and the other fundamentally perceptual (e.g., the automatic reaction to sensory inputs). This possibility would not be compatible with the sharp demarcation model (e.g., favored by Firestone and Scholl, 2016), since recognitional capacities seem to determine perceptual processing in such cases.

In what sense can preconceptual states that are not cognitively penetrable lead to attention modulation that is cognitively driven? As mentioned, one possibility is that conscious and unconscious non-conceptual states overlap systematically with contents that can be described categorically by an organism with conceptual capacities. Given the accuracy and reliability of the mechanisms that produce such preconceptual states, one could think of these states as a representational framework that structures an interface for more abstract representations. Language seems to be present only in humans, and as a matter of methodology, it is best not to attribute conceptual capacities to other species (Bermúdez, 2003, calls this a *minimalist approach* to non-linguistic thought). Taking a minimalist approach is fundamental to explain many navigational capacities that are best understood either as measurement-based representations or scenario contents. It would be inappropriate to characterize these representations in terms of language, concepts, or linguistic-propositional attitude psychology. Actually, some authors think that even in the case of propositional attitude attribution there are reasons to be skeptical about adopting a linguistic-propositional model instead of a more minimalist one (Matthews, 2007).

The proposal mentioned previously, that access consciousness may be responsible for cognitive penetration without causally and directly changing the contents of early perception (including phenomenally conscious perception), can now be spelled out in more detail. Early processing is cognitively impenetrable, intentional, and representational, and it can either be phenomenally conscious (producing experiences of a sensorial kind) or occur unconsciously—in accordance with CAD. These early perceptual states have a content that can be characterized as non-conceptual or non-propositional (for discussion of how to characterize the representational nature of these states see Montemayor, 2013). Then working memory and, in the case of humans at least, conceptual representations, can influence, guide, and indirectly determine the contents of perception at later stages. Working memory processes can also help maintain representations of task-relevant features by activating early feature selection regions of the visual cortex (Serences et al., 2009), which suggests a top-down influence on early vision activations. In fact, various studies testing the memory for sensory signals suggest that the circuitry underlying the working memory involved in these tasks includes cortical areas that do the processing of these signals (for a review, see Pasternak and Greenlee, 2005). Nevertheless, such modulations of early vision are consistent with the CAD approach. Also consistent with CAD is the indirect guidance of late perception, which may depend not only on access-conscious states with propositional content, but also on other motivational and phenomenologically powerful states, such as emotions. This is all consistent with early perception being cognitively impenetrable. But the interface between early and late perception shows that the interaction between perception and cognition is vital for concept acquisition. This clarification is important, because one way of interpreting Firestone and Scholl’s (2016) proposal is that such an interface is never possible and that there is no kind of cognitive penetration, even at *later* perceptual stages.

As a cognitive phenomenon, concept acquisition seems to critically depend on perceptual processes on some level. Fodor (1983, 1998), who is a prominent proponent of the modular and encapsulated architecture view that is putatively incompatible with penetrability, explains concept acquisition as follows: “We have the kinds of minds that often acquire *the concept X from experiences whose intentional objects are properties belonging to the X-stereotype*” (Fodor, 1998, pp.137–138; his emphasis). These properties are not based on stored memories, otherwise how could one even *acquire* a concept? What Fodor calls a ‘stereotype’ is not a judgment, but a statistical notion that captures *perceptual* regularities (Fodor, 1998, p.138). Fodor insists that perceptual experiences are necessary for concept acquisition. If only judgments were necessary for this, how could one acquire a perceptual concept in the first place? So conceptual recognition seems to be an essentially perceptual process. Even if one holds that concepts are innate, perceptual processes are still necessary to *acquire* such concepts (obviously, for those who deny innatism, perceptual processes *suffice* to explain concept acquisition). Concept acquisition is neither explicit judgment nor merely unconscious inference, and favoring a modular and encapsulated architecture (e.g., Pylyshyn, 1999, 2003) can still be compatible with having a conceptual interface between cognition and perception.

Below, we draw a distinction between linguistic labels and conceptual categories, which further clarifies the processes underlying concept acquisition. First, we want to expand on how the distinction between early and late perception relates to traditional issues in epistemology. When you see a red cup, seeing it as a cup that has the property of being red obviously means that you possess the concepts ‘red’ and ‘cup.’ But your perceptual system can be in a phenomenal state with the red cup as part of its content, independently of these concepts (as it occurs with infants, and presumably in other species). In other words, your perceptual system can have a visual experience of the red cup without seeing it as an object that falls under the category ‘red cup.’ For this reason, it seems that theories in cognitive science must allow for the distinction between non-epistemic and epistemic *seeing* (e.g., seeing a bundle of features versus seeing *something as* an instance of a conceptual category).

Cases of expertise generate an interface not only with concepts, but also with larger repertoires of judgments and beliefs. Looking out your window, you see a bird land on a nearby tree limb and you notice its gray and black colors. Your expert friend, an ornithologist, sees not only the bird and its colors, but also sees it as a hooded crow. This contrast can be interpreted in several ways: you see an object and its colors, and after attending to it carefully you see that it is a crow; or you see a bird and while you see it as a crow, your expert friend sees it as a hooded crow. In the latter case it seems clear that you and your expert friend see the same bird (but see Siegel, 2010, for the claim that these might be different perceptual experiences with different contents). In the former case you see the bird and apply the concept ‘crow.’ Other species may see the bird and be in a perceptual state that disposes the animal to behave as if it were referring to crows in particular, but without needing to be in a conceptual or propositional state. You and your friend, however, are accessing information differently even though the content of your early perceptual experiences very likely overlap. This is why access consciousness is associated with more complex forms of cognitive integration that occur at later stages of perceptual processing. You posses the concept crow and bird, but only your friend can draw the inference that this is a specific kind of crow.

Expertise (and/or prior experience) can change how we see something conceptually, at the access consciousness level, but not perceptually, at the early phenomenally conscious level. It can affect perception at later perceptual stages, as when perceptual contents are integrated with motivational states. Being an expert might help you notice the nuanced details of a bird that enable you to identify it as a certain species, compared to the naïve observer that just sees it as some kind of bird (i.e., attention to the detail might differ, though the same perceptual contents are available to both observers). Expertise could provide a form of pre-cueing effect. For example, by tuning the nervous system to integrated contents, musicians are able to respond to multisensory stimuli more efficiently (Landry and Champoux, 2017). These effects modulate or guide attention, rather than determine what one perceives by affecting how information is processed. Even in cases of sensory phenomena, such as adaptation or negative after-images, changes in perception are due to the unusual and consistent activation of visual neurons (e.g., by forcing a constant fixation, a stimulus in the periphery can disappear due to neural fatigue), and would not be considered cases of cognitive penetration. In fact, these changes in adaptation occur because gaze is directed in such a way as to induce these phenomena, which are examples of how the modules of perception can be directed in ways to exploit their inherent characteristics, and not an example of cognition directly changing the processing within the modules of early perception (see Clifford et al., 2007). These adaptation effects occur at several levels of perception that include late ones, as in the case of face perception (Webster and MacLeod, 2011). It is the modulation based on concepts and propositional content that is distinctly characteristic of access consciousness, which according to CAD, need not characterize phenomenal consciousness, including subjectively experienced adaptation effects, thereby allowing for the cognitive impenetrability of early perceptual states.

Concept acquisition begins with perceptual processes that provide contents that need not be conceptualized to be informative. Then later perceptual stages interface with conceptual information and then store categorical information into memory. Such interfaces are critical for conceptual cognition. Of course, one can combine existing concepts to form new ones independent of direct perception (e.g., a “Pegacorn” can be easily imagined if one is familiar with Pegasus and unicorns). Concept acquisition is a product of various processes, some of which are purely perceptual and others purely cognitive, and many that are a combination of the two. Partly because of this, we believe that neither the absolute impenetrability nor the pervasive penetrability views are entirely correct.

The CAD framework also allows for graded distinctions that explain why, on the one hand, early perceptual processes are so stable regardless of background beliefs and emotions, and on the other hand, why highly integrated information is susceptible to distortions based on beliefs and emotions at later perceptual stages. This is a consequence of the argument from evolution. Since intentionality evolved, an interface between cognitively penetrable perception and cognitively impenetrable perception must have evolved. The perception of magnitudes offers a particularly interesting case. Perceptually represented magnitudes for motor control and navigation (e.g., duration, distance, or rate) differ from conscious attention to the duration of sensations and emotions, including experienced effort. The former are very reliable across species while the latter are susceptible to well-confirmed distortion effects (Kahneman, 2000). Partly because of the difference in integration and susceptibility to distortion effects, there are two models in assessments of experience based on their duration or intensity: the memory-based and moment-experience based models (Kahneman, 2000, p. 692). This contrast between the early perception of magnitudes and more recent interfaces between perceptual magnitudes and conceptualized experiences has clear implications for agency and planning, and it suggests that different species must represent themselves in time differently (Montemayor, 2010).

Perhaps among the evolutionarily oldest forms of early perception is the perception of magnitudes for navigation. Perceptual capacities for navigation are among the most reliable skills that have been verified across species, including insects (Gallistel, 1990). These perceptual capacities rely on representations that are non-conceptual, and can be explained in terms of scenario content (see also Montemayor, 2013, for discussion of why these are representational). Conceptualized emotions (and their duration and intensity), however, are much more difficult to verify in other species and cannot be assumed to be present in many of them (e.g., in insects that can reliably navigate and attend to magnitudes). Presumably, species with theory of mind capacities have a more complex interface for perception, emotion, and cognition, as the distinction between empathic and nociceptive pain shows. The possibility for cognitive penetration correlates with evolutionary history, as the argument from evolution entails, and also with the cognitive integration required for accessing propositional contents. The contrast between the perceived duration of emotions and the more basic perception of magnitudes (e.g., time, distance, and rate) can easily be accommodated by the CAD framework: there is an interface for the integration between emotions and judgments concerning intensity and value at much later stages of perception, but early perceptual processing of magnitude perception is cognitively impenetrable. This guarantees reliability, as mentioned before. In humans, there is also a conceptual interface for the integration of perceptual magnitudes and non-perceptual concepts, such as mathematical concepts concerning space, time, and rate. This interface is associated with access consciousness while the interface with emotions is a combination of late conceptual perception and the phenomenology of emotions. While some studies show that magnitude judgments can be calibrated systematically (e.g., Izard and Dehaene, 2008), these would be cases of modulating the interpretation of the output rather than a cognitive penetration of the magnitude estimation mechanism itself.

An interesting consequence of the argument from evolution in the context of CAD is that competing views about concepts may be correctly describing different *levels* of perceptual processing. Conceptual structure of the kind humans have is more abstract than any set of features or simple perceptual attention routines—it has a logical structure that allows for negation, valid inference, and compositionality. Such concepts cannot be reduced to the sums of the expected probabilities of features given a perceptual scene, but the earliest, cognitively impenetrable stages may be reducible to such feature or prototype-based analysis. This leads to two further implications of the argument from evolution concerning concepts in particular. First, the higher the degree of cognitive integration and penetration, the more logical structure is needed for cognitive influence. Second, the higher the degree of inferential integration, the more abstract and amodal the concepts are. This higher-level of cognitive integration is the one typically associated with explicit judgment (i.e., explicit judgment has logical structure). This opens the possibility for different types of featured-based prototypes operating at early stages, and more characteristically abstract conceptual representations playing different roles at different interfaces, allowing for different forms of integration and de-modularization at later perceptual stages. These interfaces would be consistent with empirical findings, such as the cross-species findings on the perception of magnitudes and the findings on the distortion of duration judgments regarding emotions in humans. Finally, one finds a similar distinction between prototype-based categorization and more abstract concepts in human development (e.g., Keil, 1989). Developmental studies indicate that infants can obtain perceptual concepts before complex forms of abstract concepts (Spelke, 1988; Spelke and Kinzler, 2007; Carey, 2009). It is with this more advanced type of conceptual interface where we could find cognitive penetrability, at later stages of perceptual processing that are integrated with cognitively driven attention modulation. These interfaces are, in the very least, evidence for the interrelation between perception and cognition at later stages. Thus, postulating different types of interfaces, based on the CAD framework and the arguments from evolution, may help explain cases of cognitive penetration at later stages while preserving the cognitive impenetrability of early perception, striking a balance between the prevailing opposite views.

## Cad as a Framework of Distinctions for Emotion, Perception, and Judgment

Emotions complicate the picture considerably. They are an important aspect of social cognition and interactions, particularly in terms of developing empathy and helping to understand others. For such reasons, emotional processing must be an integral component of human perception and cognition. Newen (2016), for instance, argues that emotions can be perceived similar to the way perceptual features are perceived. Studies suggest that emotions can be recognized in the same way as pattern recognition in other sense modalities, driven by evolutionary necessity and requiring an interaction of bottom-up and top-down processes (see Newen, 2016). Similarly, socially relevant information seems to be processed automatically, thus calling into question whether perception should include attention to social cues (Neufeld et al., 2016). If it is true that emotions and socially relevant information are processed like perceptual features, this view would strongly favor a very robust kind of cognitive penetration because we not only see the basic perceptual constancies that ground object- and feature-based attention, but also emotional and socially relevant content. In other words, if this view is correct, then emotional and social beliefs would determine a substantial portion of perception. It is important to notice that even if this were the case, it would still be compatible with early perception being cognitively impenetrable.

The main problem, however, is that this example of penetrability could simply mean guidance. There is good reason to believe that the neural systems that support emotion overlap with cognition (Pessoa, 2008), and emotional states may be considered a form of pre-cueing. For example, an emotional state, like fear, can bias how one directs attention (e.g., to more threatening aspects of environment) and thus improve interacting with the environment (LeDoux, 2012). This ability also includes non-conscious perception of emotional stimuli (see Tamietto and de Gelder, 2010). If these pre-cuing effects are very robust and systematic, there is a very clear sense in which they determine what one perceives, thus favoring some level of penetrability at later stages of processing.

Just how powerful, exactly, can cognitive penetration be in the case of emotions without being cognitively pernicious (e.g., by altering too much the contents of perception and rendering crucial perceptual invariances unstable and unreliable)? CAD also helps elucidate this issue. Emotions have an enormous impact on conscious awareness, but this impact need not be either fully perceptual or inferential. We believe this is a significant source of confusion. Emotions have a significant impact on an individual’s overall phenomenology, but having too much impact on awareness can distract from or may even suppress what one *perceives*. In such cases, the phenomenon is one of interference or hindrance of perception rather than a determination of perception (e.g., as with post-traumatic stress disorder). In other cases it may enrich perception—not by determining it, but by adding vivacity to the *overall* phenomenological experience. Aesthetic experiences and the vivacity of certain autobiographical memories are good examples of this phenomenon (Montemayor and Haladjian, 2015, pp.150–165). All these cases are best understood as late perceptual cognitive penetration (perhaps *motivational* penetration is a better term), rather than cognitive penetration of early perception (for instance, early vision).

Color perception further elucidates the importance of CAD to rigorously define cases of cognitive penetration at later stages of perceptual processing from cognitively impenetrable early vision. Color perception involves two distinct neural circuits, one for color detection and another one related to circadian regulation and emotion (Pauers et al., 2012). Do we perceive emotions when we perceive color? This does not seem plausible. Rather, we detect and experience color in early vision, and we also experience a complex state of perceptual and emotional contents at later stages of processing. Even in the case of an individual’s memory of an object’s expected color, which can influence the perceived color appearance of an object (see Hansen et al., 2006), such findings do not conclusively indicate cognitive penetration of early visual perception, but rather the stage that includes the interpretation of the signals from early vision. To complicate things further, some aspects of feature-detection may occur outside consciousness—they are mostly independent in their neural correlates (Koch and Tsuchiya, 2012). Priming of color can occur at higher levels of processing even without conscious perception of the color, as in studies that use backward masking to test priming of responses to colors that are not consciously seen (Norman et al., 2014). To accommodate this fact we need a graded framework like CAD rather than a sharp distinction between cognition and perception or a pervasive form of cognitive penetration. Color detection and color-based emotions do interact systematically at the later stages of perceptual processing that are also phenomenally conscious, but this does not entail that emotion penetrates color detection or early visual color experiences.

Regarding the cases of automatic social detection (e.g., Neufeld et al., 2016), these could be similar to detection patterns associated with social planning routines that operate independently of experienced emotions and feelings. Thus, based on CAD, it is not so easy to say that emotion is detected as part of perception, because such routines could be modeled either as unconscious processing or as specific attention routines triggered by specific perceptual conceptualized contents, rather than being constitutive of early perception, since this detection is not altered by overall phenomenology (a point entirely analogous to the distinction between magnitudes and emotion intensity mentioned above). Generally, it may be that such pattern detection of social cues would not entail systematic penetrability because they may actually occur at late levels of cognitive processing or not be perceptual at all (e.g., they could be inferential or strictly mnemonic).

The CAD framework can explain many changes in perception at later stages while justifying the impenetrability of early perception. Merely appealing to phenomenology and how switching from one attention task to another vary what one experiences does not suffice to prove penetrability precisely because of the distinctions based on the levels of CAD. Moreover, even the phenomenology of perception favors stability and continuity in experience, rather than variability caused by constant cognitive penetration. For example, as one moves around a room, the experienced color and shape constancies of the walls and furniture remain the same despite the many inferential triggers, actual and potential, that one has at any single moment. Strikingly, this also seems to be the case in dreams where there is a generally coherent experience, no matter how absurd it may be. Therefore, appeals to conscious experience may not provide decisive evidence for cognitive penetration because overall phenomenology depends on cognitive integration at late stages of perceptual processing in a way that is compatible with early perceptual impenetrability. What one needs to show in order to verify radical and pervasive cognitive penetration is that cognition determines perception at an essential level, at the earliest stages, causing changes in perception in a direct way. CAD shows that the evidence can be understood in a way that avoids this interpretation because CAD demonstrates that cognition and perception can be independent and yet interact in systematic ways. In particular, concept acquisition of basic perceptual categories is a good place to identify clear cases of cognitive penetration beyond the initial stages of early perception.

## Cognitively Driven Attention: Feature-Based, Syntactic, and Semantic

It is important to restate why resisting pervasive cognitive penetration is not only plausible because of the argument from evolution, but also as a general theoretical commitment. One reason is the problem of the impossibility of common ground among perceivers. If there is no common ground, how can one explain reliable coordination among multiple subjects for motor control and attentional tasks (e.g., that are executed when playing team sports)? One solution, offered by CAD, is that while there are significant levels of cognitive penetration at highly integrated levels of cognition and perception, there is no cognitive penetration at early conscious and unconscious perception. But it is also important to explain how exactly top-level processes influence perceptual experience. This is what CAD allows for: cognitive impenetrability of early processing with rich influence from cognition at *higher* levels of cognitive integration (e.g., attention to the intensity of emotions, or the importance of an autobiographical memory), which correspond to more evolutionary recent types of attention (for a criticism against the view that top-down pathways entail cognitive penetration, see Raftopoulos, 2001a,b).

There are several possible areas of higher-level cognition that could be susceptible to cognitive penetration. According to CAD, phenomenal consciousness varies systematically with emotional and background knowledge contents—it is *empathically* structured (Montemayor and Haladjian, 2015). How susceptible the more semantic aspects of the mind are to inference and emotional influence may depend on the *concepts* a species has and the degree of information integration—hence the importance of concept acquisition. To repeat, early perception is cognitively impenetrable, which allows for reliable and predictable motor control and coordination with external objects. These contents are processed independently of the empathic and integrative influences of cognition and emotion. This structural requirement is related to adaptive necessity, and likely appeared in other species that are evolutionarily close to humans (Zentall, 2005). Furthermore, human-like conscious awareness seems dependent on a global functional connectivity among brain modules (Wu, 2014; Godwin et al., 2015), and this may indicate a form of penetrability at later, more integrated stages of perception.

At the early stages, perceptual features are processed independently, with minimal top-down modulation, in order to reliably and accurately structure the perceptual scenes (e.g., auditory or visual scenes). This representational scaffolding supports later cognitive guidance and can be characterized as scenario content or preconceptual sensorial representation. Then, at later stages that likely depend on the intervention of working memory, feature based-attention can be guided and oriented by cognitively driven forms of attention that highlight some perceptual features and suppress or inhibit others based on cognitive and motivational information. Some of these cognitively driven types of attention likely evolved at different times. Some of them modulate detection; others exclusively concern conceptual information and can only be found without controversy in humans. The range of influence of cognition on perception is quite vast and it increases with the degree of cognitive integration, characteristic of late perceptual processing. According to CAD, there is a type of attention at late stages of integration that is fully independent of specific perceptual experiences and that is exclusively driven toward access to propositional contents. We have argued that this kind of cognitively driven attention plays an important role in specifying the contents of late perception, but that it cannot directly change early perception, including the perceptual experiences associated with early stages of perception.

The consciousness and attention dissociation also helps address the previously mentioned difficulty that perception may occur outside consciousness, independently of whether or not the contents of perception are susceptible to cognitive penetration. Consider the result by Vishton et al. (2007) concerning the Ebbinghaus illusion, in which the instruction to grasp the stimulus reduces the illusion. This effect of a reduction in the illusion was found in previous studies where acting on a stimulus producing an illusion indicated a more accurate internal representation than what was consciously perceived. That is, while the phenomenology of perception is tricked by an illusion (e.g., the Müller-Lyer illusion), perception for action is not (Stöttinger and Perner, 2006). Also, this kind of performance can be affected by emotional states (van Ulzen et al., 2008), which indicates that emotion can influence conscious experience, but only at a higher level of integration and processing, as the argument from evolution entails. Similarly, desiring something might affect how it is consciously perceived; for example, an appealing location might seem closer than an unappealing one that is at the same physical distance from the observer (Alter and Balcetis, 2011). Such studies are examples of how conscious perceptions may be affected by certain mental states, and that there can be a dissociation between the information that enters awareness and the unconscious information used for other perceptual processes. It is unlikely, however, that these effects could influence the perceptual-navigational system (e.g., the system we use to walk across a room), or the experiences produced by early perception.

Another example of how feature detection in conscious perception can differ from that used to execute motor actions is seen in an experiment investigating the double-drift illusion. This illusion occurs when an object moves in the periphery of the visual field along a specific trajectory, but because the object has a texture that moves orthogonal to this trajectory, the overall perceived movement of the object does not correspond to the veridical path. In other words, an illusory path is perceived because of the combination of motion information from the internal motion of the object as well as its actual trajectory. In a recent study by Lisi and Cavanagh (2015), participants were asked to make an eye movement to one of these moving objects (that disappeared as soon as the eye movement began), and they found that the eyes landed closer to the veridical path as opposed to the perceived illusory path. This suggests that the information sent to the motor system is not susceptible to the illusion, since the motor system can execute correct eye movements, even though the illusion is consciously perceived. An implication of these results is that unconscious perception can be highly accurate as well as integrated with cognitive-driven goals.

The CAD framework also allows for a more useful distinction that can potentially clarify ambiguities. Consider Kravitz and Behrmann’s (2011) finding concerning facilitation by a concept: faster response times to detect ‘h’ based on prior exposure to ‘H.’ This kind of cognitively driven attention to syntactic features should not be considered cognitive penetration. For similar reasons, semantic priming should also be considered an attentional effect that is cognitively driven and that occurs at later stages of processing. In the evolution of the visual and other perceptual systems, it is likely that feature-based attention and basic forms of object-based attention evolved first, and only later can one find complex forms of semantically driven attention to features relevant to expertise and propositional contents (see Haladjian and Montemayor, 2015). Attention based on propositional content is, therefore, a kind of cognitive guidance that must occur at later stages of perceptual processing and which must have evolved more recently. This kind of cognitive guidance at later stages can influence inference, memory, object recognition, and concept categorization. In the case of human cognition and perception, this kind of cognitively driven attention to semantic contents is the most important component that facilitates a powerful interface between cognition and perception, and it provides the basic scaffolding for concept acquisition of all kinds. As mentioned before, concept acquisition allows for many kinds of cognitive penetration at later stages of processing, and it is fundamental to understand human perception.

There is yet another, and perhaps even more recent, kind of cognitively driven attention that modulates late perceptual contents: attention to syntactically structured perceptual patterns. The complex hierarchical structure of human language must be somehow perceived. The question is exactly how. If Berwick and Chomsky (2016) are right, the capacity to detect syntactic patterns evolved quite recently in our species. In fact, if it is true that the capacity to articulate and combine strings of symbols hierarchically is as recent as 200,000 to 150,000 years ago (Berwick and Chomsky, 2016, p. 54, indicate that it is only 60,000 years ago that it certainly emerged), then it must be one of the *most recent* events in our cognitive evolution. While syntax processing has a very significant impact on human cognition, it need not operate by constantly influencing what we perceive (unlike conceptually based late perception, which is essential for epistemic seeing and epistemic perception more generally). Rather, it may operate in the way motor control operates: in a highly automatic and reliable fashion that cannot be made explicit through discursive judgment and which processes information beyond conscious access. If so, even in spite of its very recent evolution, syntax processing may not provide a robust interface for cognition and perception, and interesting cases of cognitive penetration at late perceptual stages may be limited to semantic processing. This is an issue that needs to be studied in more detail.

This brings us to the last point we want to make. The fact that perception is stable and invariant at the early stages supports not only our cognitive systems but also motor control and action. Early perception plays the critical role of making this possible, by not allowing direct casual influences from cognition or emotion on the processing of the most basic stages of perceptual scene structuring. Basic perceptual experiences are also stable in this way and, moreover, they are experienced in a way that does not necessitate conceptual or propositional guidance. Conscious *access* to contents, on the other hand, likely requires a high level of integration of information within the brain, which is the argument made by global workspace theories of consciousness (e.g., Dehaene and Naccache, 2001; Baars, 2005), with increased functional connectivity among different neural modules (rather than within modules) being associated with such conscious awareness (Godwin et al., 2015). Cognitive penetration is likely to be found at these later stages of perceptual processing, and crucially, at the interface between early contents and different forms of concept formation. A further question is the extent to which empathic and motivational effects guide late perception. With the rich conceptual framework of human cognition, the interface between emotion, cognition, and perception allows for many kinds of cognitive penetration at these later stages of processing. Semantic and syntactic guidance through cognitively driven attention is a critical part of this process.

Acquiring concepts does not *directly* affect how features are detected at the earliest level, but they do determine what we epistemically perceive (e.g., as a member of a category). Having a specific concept is not as urgent as responding to a feature essential for survival, but basic categorization, even if it is of a preconceptual kind, can help in urgent situations, as the alarm calls of some animals show. It can also lead to complex forms of planning, mental travel, and even theory of mind, as the quasi-conceptual capacities of birds demonstrate. Fully fledged concepts, as found in humans, lead to a cognitive framework that allows not only for epistemic seeing, but also for inferential judgment (including discursive inference), and epistemic justification. Based on CAD and the argument from evolution, it is useful to think of these capacities as falling under different levels of cognitive integration at higher-levels of perceptual information processing. To reiterate, this is all compatible with the cognitive *im*penetrability of early perception.

Thus, CAD helps clarify how the fact that perception is deeply related to cognition and emotion is compatible with the cognitive impenetrability of early perceptual processing. Conceptual interfaces are at the center of the relation between cognition and emotion. These conceptual interfaces manifest in forms of perceptual pre-cuing, biases, modulation, and guidance through the mechanism of cognitively driven attention. These interfaces also provide the framework for the type of consciousness associated with access to propositional contents, which according to CAD, is dissociated from the experiences produced by early phenomenally conscious perception. Early perception guarantees stability and reliability, as well as a perceptual common ground with other organisms. Late perceptual processing provides a rich framework of possibilities that enrich perception in many ways. Finally, semantic and syntactic influences in late perception increase these possibilities in ways that cannot be found in any other species, and makes human perception the rich manifold of contents that make possible the very complex behavior that characterizes humanity.

## Conclusion

How the world appears to us can depend largely on our expectations, beliefs, and desires. The debate on cognitive penetration has explored this issue in the last few decades from different perspectives, particularly those concerning cognitive architecture and semantic content. The conclusion many authors reach is that cognitive penetration is either largely pervasive or inexistent. We argue that a more nuanced perspective is required. The CAD framework allows for such a perspective, informed by findings from the research on consciousness and attention, and their evolution. More specifically, CAD helps explain why although there may be many cases of cognitive penetration in late perception, early perception must be cognitively impenetrable.

With the CAD framework, a more balanced approach to cognitive penetration is feasible. An interesting question is: could a similar balance be achieved without it? We cannot explore this issue in detail here, but we believe that at the very least, CAD is the *best* way to achieve this balance. It may be the *only* way to achieve such a balance in a rigorous way, but we will not argue for this stronger claim here. However, we leave this consideration in favor of CAD: the evidence, including evolution, does not support as strongly an interface without CAD. For instance, such an interface could concern only unconscious processing (e.g., constituted by Helmholtzian inferential abilities). Alternatively, this interface could involve exclusively conscious information, requiring subjectively experienced integration for any perceptual process. The evidence indicates that neither of these options is likely true. Thus, the interface between cognition and perception seems to be fundamentally structured in terms of CAD.

Given the implications of CAD and the argument from evolution, we argued that concept acquisition is a particularly important topic with respect to cognitive penetration, with ramifications for the integration of emotions, inferential reasoning, and recognitional processes. Perception and cognition may be largely independent, and they are fully independent at early stages, but there are systematic ways in which they interact. The more cognitive integration there is, the more cognitive penetration one finds. Perhaps, as suggested above, there may even be more than one interface for cognitive penetration because there are many kinds of cognitive modulation in late perception. Yet despite this systematic interaction between cognition and perception at such late stages, cognitive penetration is not pervasive.

Besides providing positive suggestions for addressing the problem of penetrability in a more thorough theoretical way, this paper also raises challenging questions. What kind of conceptual or epistemic capacities underlie different forms of penetrability? Which capacities necessitate cognitive penetration? How can one verify such capacities across different species? How is it possible to integrate the findings on consciousness and attention, as well as their dissociation, in a way that addresses the problem of cognitive penetration? The findings on animal cognition and future research on how our own capacities compare to those of other species, particularly in the development of semantic and conceptual guidance, is fertile ground for exploration. The argument from evolution, especially as it concerns the development of different forms of intentionality, should help guide future investigations in this area.

## Author Contributions

All authors listed, have made substantial, direct and intellectual contribution to the work, and approved it for publication.

## Conflict of Interest Statement

The authors declare that the research was conducted in the absence of any commercial or financial relationships that could be construed as a potential conflict of interest.
